# Observation of electrochemically generated nitrenium ions by desorption electrospray ionization mass spectrometry†
†Electronic supplementary information (ESI) available: MS/MS data for structural confirmation and experimental procedure details. See DOI: 10.1039/c5sc02939b



**DOI:** 10.1039/c5sc02939b

**Published:** 2015-10-08

**Authors:** Timothy A. Brown, Niloufar Hosseini-Nassab, Hao Chen, Richard N. Zare

**Affiliations:** a Department of Chemistry , Stanford University , Stanford , CA 94305-5080 , USA . Email: zare@stanford.edu; b Center for Intelligent Chemical Instrumentation , Department of Chemistry and Biochemistry and Edison Biotechnology Institute , Ohio University , Athens , OH 45701-2979 , USA . Email: chenh2@ohio.edu

## Abstract

We report the observation of the electrochemically generated nitrenium ions of 4,4′-dimethyoxydiphenylamine and di-*p*-tolylamine in solution by mass spectrometry. This setup takes inspiration from desorption electrospray ionization mass spectrometry to sample directly from the surface of a rotating waterwheel working electrode for mass spectrometric analysis. Detection of the 4,4′-dimethyoxydiphenylamine nitrenium ion was expected based upon *para*-methoxy resonance stabilization, whereas observation of the di-*p*-tolylamine nitrenium ion might be unexpected because resonance stabilization from the *para*-substituted position is unavailable. However, the short timescale analysis of the setup allows for the isolation of the di-*p*-tolylamine nitrenium ion, which is electrogenerated in solution and detected mass spectrometrically.

## Abstract 2

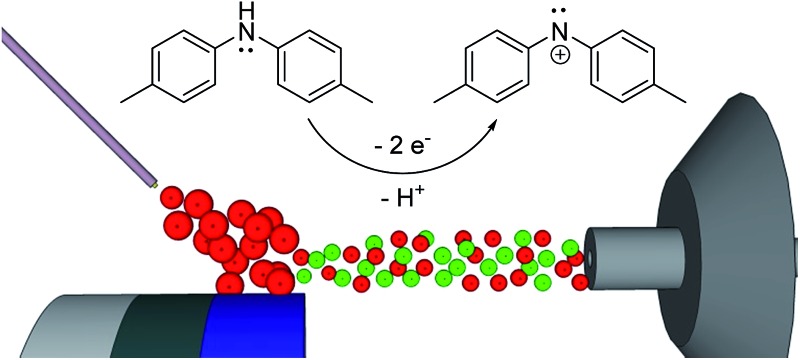
Electrochemically generated nitrenium ions from amines can be detected mass spectrometrically using desorption electrospray ionization on a rotating conducting wheel covered with an electrolyte film.

## Introduction

Nitrenium ions are reactive cation intermediates that are isoelectronic to carbenes.^1–3^ Understanding nitrenium ions impacts not only organic synthesis but also has biological significance due to their reactivities toward nucleobases.^4–7^ Nitrenium ions can be formed in a number of ways including chemically,^4,8^ photochemically,^9–11^ and electrochemically.^12–15^ Arylamines have been studied as model systems for formation of the fleeting nitrenium species in solution.^8–14,16^ Mass spectrometry (MS) has been utilized to observe nitrenium ions before *via* ion/molecule reactions,^4,8,17,18^ however the mass spectrometric characterization of these arylamine nitrenium intermediates from electrochemical generation has been scarce outside of the observation of the nitrenium ion of clozapine.^7,15,19^ Herein we present the first application of desorption electrospray ionization (DESI)^20,21^ MS in detecting nitrenium ions generated electrochemically from arylamines.

## Experimental

**Fig. 1:**
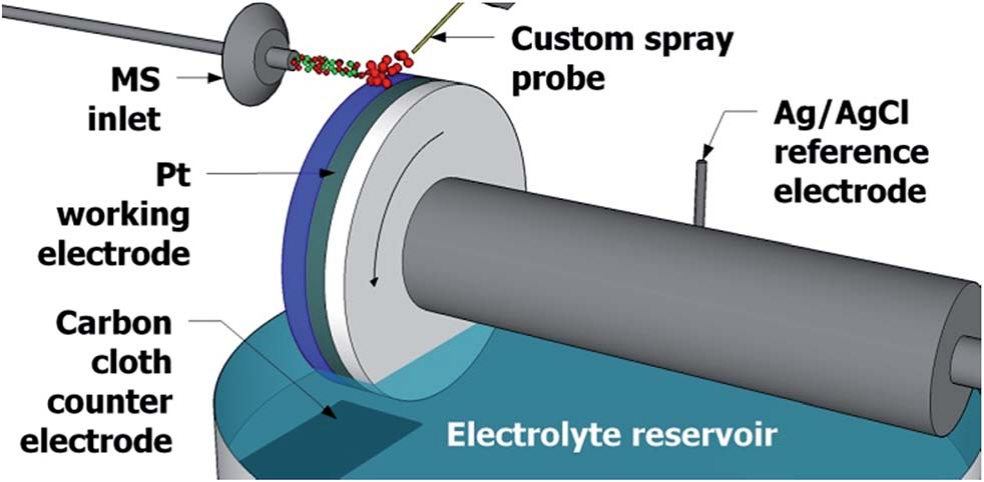
The setup used for detection of electrochemical reaction intermediates, employing a rotating waterwheel electrode. Wheel rotation is indicated by the curved arrow.

The experimental design in Fig. 1 greatly resembles the waterwheel setup previously reported,^22,23^ which employs a round rotating platinum working electrode immersed in an acetonitrile solution containing 1 mM lithium triflate as the electrolyte. The distance between the MS inlet and the working electrode surface is approximately 2 mm. As the working electrode rotates, a thin layer of liquid film develops on the electrode surface (approximately 1 mm in thickness). A plain carbon cloth counter electrode and an Ag/AgCl reference electrode are immersed in the reservoir of electrolyte solution. A metal contact (not shown) rests against the platinum working electrode to complete the three-electrode system. A potentiostat (WaveNow, Pine Research Instrumentation, Durham, NC) is used to apply a potential across the three electrodes.

Above the rotating waterwheel system a custom spray probe directs a stream of sample microdroplets to the surface of the working electrode. Studies have shown that electrospray ionization and DESI sources can function as an electrochemical cell^24–27^ and so no high voltage is applied to the sample spray to minimize in-source oxidation. The spray droplets hit the surface of the thin film of electrolyte solution, now thinner than 1 mm due to the N_2_ nebulizing gas pressure, on the surface of the working electrode. Much like in DESI-MS and easy ambient supersonic spray^28–30^ MS, tinier secondary microdroplets are directed into the mass spectrometer^31,32^ and are analyzed with an LTQ Orbitrap XL hybrid mass spectrometer (Thermo Fisher Scientific, San Jose, CA) where the *m*/*z* ratio is determined utilizing the high mass accuracy and high resolving power of the Orbitrap mass analyzer.^33^

## Results and discussion

### Electrogeneration of DMDPA nitrenium ion

**Fig. 2:**
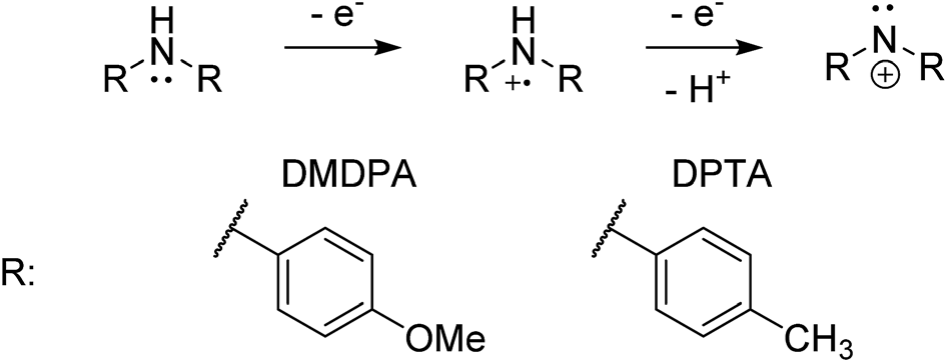
The general scheme for electrochemical generation of nitrenium ions from secondary arylamines.

**Fig. 3:**
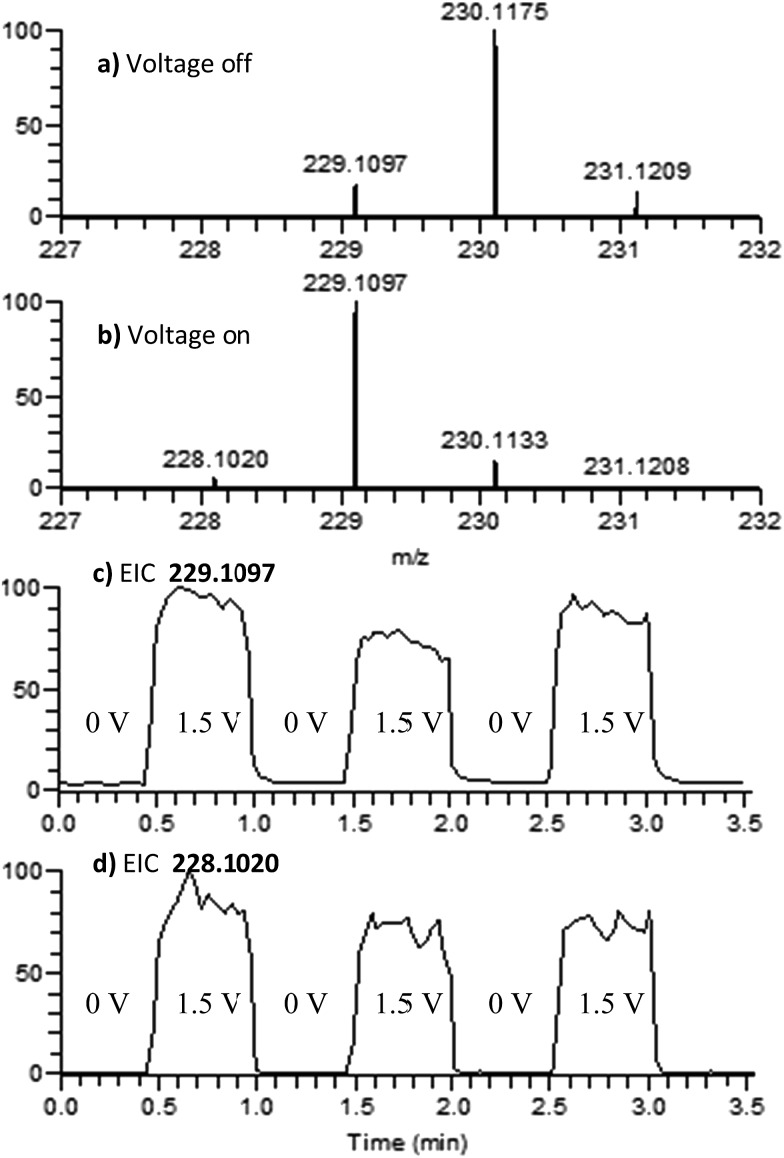
Positive-ion mode mass spectra of DMDPA. (a) 0.0 V applied to the working electrode; (b) 1.5 V applied to the working electrode; (c) EIC for the 229.1097 peak as a function of the applied potential; and (d) EIC for the 228.1020 peak as a function of the applied potential.

4,4′-Dimethoxydiphenylamine (DMDPA) was chosen as a model system because nitrenium formation from DMDPA has been extensively studied.^14,16^ DMDPA is proposed to be electrochemically oxidized to the nitrenium ion by losses of two electrons and one proton, passing through a radical cation intermediate (Fig. 2). In this experiment, 100 μM of DMDPA was prepared in 1 mM solution of lithium triflate (LiOTf) in acetonitrile. When the analyte solution is sprayed at an injection flow rate of 10 μL min^–1^ onto the working electrode rotating at 1.0 rev per s, *m*/*z* 230.1175 is observed, ascribed to the protonated DMDPA cation (Fig. 3a, theoretical *m*/*z* 230.1176, error –0.2 ppm) as well as *m*/*z* 229.1098, attributed to the DMDPA radical cation (Fig. 3a, theoretical *m*/*z* 229.1097, error 0.0 ppm). When an oxidation potential of 1.5 V is applied across the rotating working electrode the intensity of 229.1097 increases almost ten-fold (Fig. 3b), suggesting that the DMDPA radical cation is formed through electrochemical oxidation of DMDPA. A peak at *m*/*z* 228.1020 is also observed, which is ascribed to the DMDPA nitrenium ion (Fig. 3b, theoretical *m*/*z* 228.1019, error +0.5 ppm). Upon CID, the DMDPA nitrenium ion gives rise to fragment ions of *m*/*z* 213 and 197, corresponding to the losses of CH_3_ and CH_3_O radicals, respectively, consistent with its structure (Fig. S3, ESI†). The MS signals of the DMDPA radical cation and DMDPA nitrenium ion increase greatly when an oxidizing potential is applied to the working electrode, as is observed in the extracted ion chromatograms (EIC) at specific *m*/*z* values (Fig. 3c and d). This indicates that these species are formed *via* electrochemical oxidation of DMDPA and is the first electrogenerated nitrenium ion observed with DESI-MS.

### Electrogeneration of DPTA nitrenium ion

The electrochemical oxidation of di-*p*-tolylamine (DPTA) was also studied. DPTA is proposed to undergo nitrenium formation by the same pathway as DMDPA (Fig. 2), but this system lacks the *para*-methoxy groups that can stabilize both the radical cation and nitrenium species through resonance. Because of this the half-life time of these species in solution is estimated to be shorter compared to corresponding DMDPA intermediates.

**Fig. 4:**
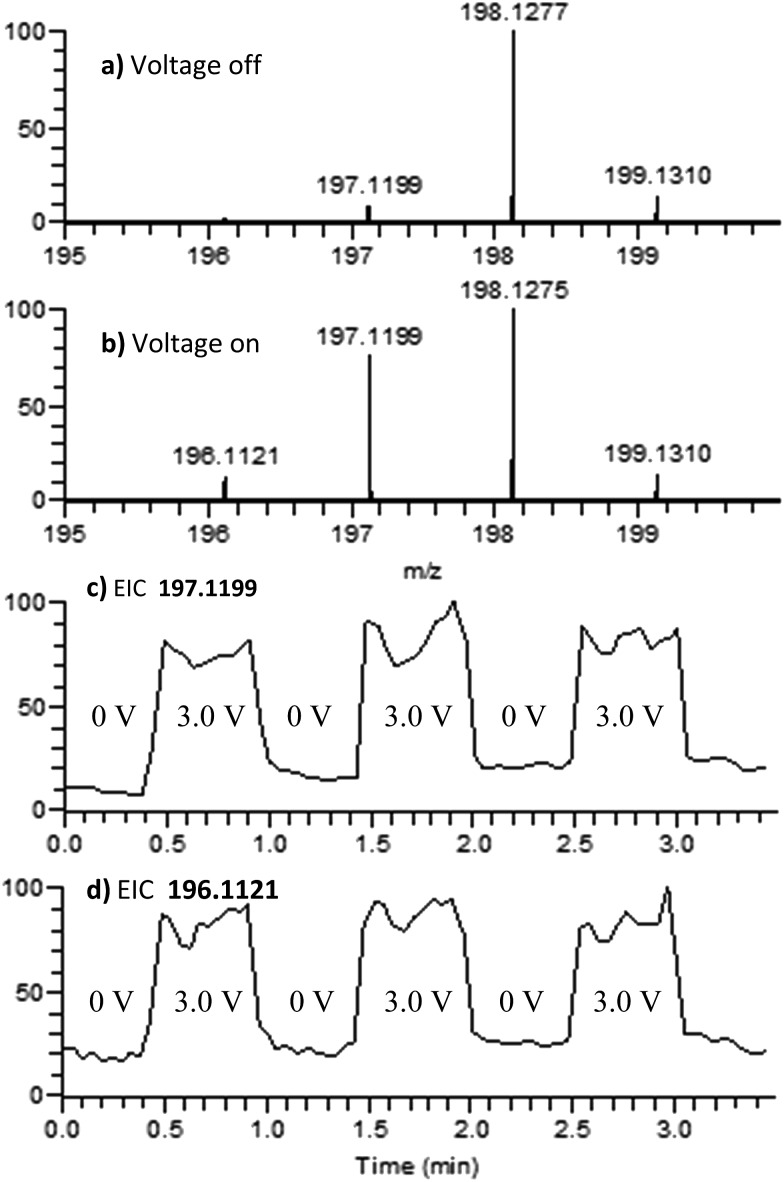
Positive-ion mode mass spectra of DPTA. (a) 0.0 V applied to the working electrode; (b) 3.0 V applied to the working electrode; (c) EIC for the 197.1199 peak as a function of the applied potential; and (d) EIC for the 196.1121 peak as a function of the applied potential.

100 μM of DPTA was prepared in 1 mM solution of LiOTf in acetonitrile. When the analyte solution is sprayed at an injection flow rate of 10 μL min^–1^ onto the working electrode rotating at 1.0 rev per s, a mass peak at *m*/*z* 198.1275 is observed, ascribed to the protonated DMDPA cation (Fig. 4a, theoretical *m*/*z* 198.1277, error –0.9 ppm). When an oxidation potential of 3.0 V is applied across the rotating working electrode the emergence of *m*/*z* 197.1199, corresponding to the DPTA radical cation (Fig. 4b, theoretical *m*/*z* 197.1199 error 0.0 ppm), is suggested to arise from electrochemical oxidation of DPTA. The increase in the signal intensity of 196.1121, believed to be the DPTA nitrenium ion (Fig. 4b, theoretical *m*/*z* 196.1121, error 0.0 ppm) is also inferred to stem from the electrochemical oxidation of DPTA. For DPTA, the *para*-methyl groups provide hydrogens that are available for elimination, leading to the formation of a constitutional isomer of the desired amine. Performing the analysis on the analogous d-14 labelled DPTA determined that both the nitrenium ion and the elimination product are formed as a function of an oxidative potential and is discussed further in the ESI.†

Upon CID, the DPTA nitrenium ion gives rise to a fragment ion of *m*/*z* 181 by loss of a CH_3_ radical, consistent with its structure. The MS signals of the DPTA radical cation and DPTA nitrenium ion increase greatly when an oxidizing potential is applied to the working electrode, as is observed in the extracted ion chromatograms (Fig. 4c and d). This observation indicates that these species are formed *via* electrochemical oxidation of DPTA.

## Conclusions

The results presented here show evidence for the electrochemical generation of nitrenium ions through the proposed radical cation intermediate. The short timescale of electrogeneration and isolation allows for the observation of the reactive intermediates. This study emphasizes the power of DESI-MS as an analytical tool in identifying reactive intermediates in electrochemical processes.

## References

[cit1] Falvey D. E., in Reactive Intermediate Chemistry, ed. R. A. Moss, M. S. Platz and M. Jones Jr, John Wiley & Sons, Inc., 2003, pp. 593–650.

[cit2] Gassman P. G. (1970). Acc. Chem. Res..

[cit3] Borodkin G. I., Shubin V. G. (2008). Russ. Chem. Rev..

[cit4] Liu Z. C., Uetrecht J. P. (1995). J. Pharmacol. Exp. Ther..

[cit5] Maggs J. L., Williams D., Pirmohamed M., Park B. K. (1995). J. Pharmacol. Exp. Ther..

[cit6] Williams D. P., Pirmohamed M., Naisbitt D. J., Maggs J. L., Park B. K. (1997). J. Pharmacol. Exp. Ther..

[cit7] Lohmann W., Hayen H., Karst U. (2008). Anal. Chem..

[cit8] Chen H., Chen H., Cooks R. G., Bagheri H. (2004). J. Am. Soc. Mass Spectrom..

[cit9] Chiapperino D., McIlroy S., Falvey D. E. (2002). J. Am. Chem. Soc..

[cit10] Moran R. J., Cramer C., Falvey D. E. (1997). J. Org. Chem..

[cit11] Thomas S. I., Falvey D. E. (2007). J. Org. Chem..

[cit12] Rieker A., Speiser B. (1991). J. Org. Chem..

[cit13] Genies E. M., Lapkowski M. (1987). J. Electroanal. Chem. Interfacial Electrochem..

[cit14] Serve D. (1975). J. Am. Chem. Soc..

[cit15] van Leeuwen S. M., Blankert B., Kauffmann J.-M., Karst U. (2005). Anal. Bioanal. Chem..

[cit16] Svanholm U., Parker V. D. (1974). J. Am. Chem. Soc..

[cit17] Yurkovich M. J., Duan P., Shea R. C., Watkins M. A., Mandell S. M., Tippmann E. M., Jason Li S., Platz M. S., Kenttämaa H. I. (2009). Int. J. Mass Spectrom..

[cit18] Chen H., Zheng X., Yang P., Cooks R. G. (2004). Chem. Commun..

[cit19] van den Brink F. T. G., Büter L., Odijk M., Olthuis W., Karst U., van den Berg A. (2015). Anal. Chem..

[cit20] Takáts Z., Wiseman J. M., Gologan B., Cooks R. G. (2004). Science.

[cit21] Takáts Z., Wiseman J. M., Cooks R. G. (2005). J. Mass Spectrom..

[cit22] Brown T. A., Chen H., Zare R. N. (2015). J. Am. Chem. Soc..

[cit23] Brown T. A., Chen H., Zare R. N., Brown T. A., Chen H., Zare R. N. (2015). Angew. Chem., Int. Ed..

[cit24] Abonnenc M., Qiao L., Liu B., Girault H. H. (2010). Annu. Rev. Anal. Chem..

[cit25] Prudent M., Méndez M., Roussel C., Su B., Lion N., Rossier J. S., Girault H. H. (2009). Chim. Int. J. Chem..

[cit26] Van Berkel G. J. and Kertesz V., in Electrospray and MALDI Mass Spectrometry, ed. R. B. Cole, John Wiley & Sons, Inc., 2010, pp. 75–122.

[cit27] Benassi M., Wu C., Nefliu M., Ifa D. R., Volný M., Cooks R. G. (2009). Int. J. Mass Spectrom..

[cit28] Haddad R., Sparrapan R., Kotiaho T., Eberlin M. N. (2008). Anal. Chem..

[cit29] Haddad R., Milagre H. M. S., Catharino R. R., Eberlin M. N. (2008). Anal. Chem..

[cit30] Fernandes A. M. A. P., Fernandes G. D., Barrera-Arellano D., de Sá G. F., Lins R. D., Eberlin M. N., Alberici R. M. (2014). J. Mass Spectrom..

[cit31] Costa A. B., Graham Cooks R. (2008). Chem. Phys. Lett..

[cit32] Costa A. B., Cooks R. G. (2007). Chem. Commun..

[cit33] Perry R. H., Cooks R. G., Noll R. J. (2008). Mass Spectrom. Rev..

