# Supplementary material for: Observation of electrochemically generated nitrenium ions by desorption electrospray ionization mass spectrometry
Source: Chem Sci. 2015 Oct 8;7(1):329–32. doi: 10.1039/c5sc02939b (PMC5518571; doi:10.1039/c5sc02939b)
Supplement: Supplementary file 1 [file SC-007-C5SC02939B-s001.pdf]

Electronic Supplementary Material (ESI) for Chemical Science.
This journal is © The Royal Society of Chemistry 2015

Supporting Information
Observation of Electrochemically Generated Nitrenium Ions by Desorption Electrospray Ionization Mass Spectrometry

Timothy A. Brown‡, Niloufar Hosseinei Nassab‡, Hao Chen†*, Richard N. Zare‡*

‡Department of Chemistry, Stanford University, Stanford California

†Center for Intelligent Chemical Instrumentation, Department of Chemistry and Biochemistry and Edison Biotechnology Institute, Ohio University, Athens, OH

Corresponding Authors:
Richard N. Zare, Tel: 650-723-3062
Email: zare@stanford.edu
Hao Chen, Tel: 740-593-0719
Email: chenh2@ohio.edu

Table of Contents

PAGE

General Information S2

Instrumentation S2

Chemicals S2

Electrochemical Methods S2

CID MS/MS spectra of protonated 4,4'-dimethyoxydiphenylamine S3

CID MS/MS spectra of 4,4'-dimethyoxydiphenylamine radical cation S3

CID MS/MS spectra of 4,4'-dimethyoxydiphenylamine nitrenium ion S4

CID MS/MS spectra of protonated di-p-tolylamine S4

CID MS/MS spectra of di-p-tolylamine radical cation S5

CID MS/MS spectra of di-p-tolylamine nitrenium ion S5

Synthesis of d-14 di-p-tolylamine S6

Determination of di-p-tolylamine ECMS product S7

General Information. All of the preparations, manipulations, and electrochemical analyses were carried out under atmospheric pressure conditions. Reagents and solvents were purchased from commercial sources and were used without further purification.

Instrumentation. All MS data were acquired on an LTQ Orbitrap XL hybrid mass spectrometer (Thermo Fisher Scientific, San Jose, CA, USA). Analyte solutions were infused through a fused silica capillary tubing (50 µm i.d., 150 µm o.d.) surrounded by a stainless steel capillary (250 µm i.d.), that directed the nebulizing N_2 gas, using a custom-built source. The source is positioned ~2 mm above the surface. The rotating working electrode is positioned ~3 mm from the inlet of the mass spectrometer. The Orbitrap ion transfer capillary was held at 275°C for all experiments. The resolution was set to 60,000 at *m/z* = 400 for all analyses except the d-14 labelled DPTA experiment, where the resolution was set to 100,000 at *m/z* = 400. A small amount of tetrabutylammonium hexafluorophosphate was dissolved in the electrolyte solution to be used as an internal mass standard during data analysis. All data were analyzed using the Qual Browser feature of the Xcalibur™ program (Thermo Fisher Scientific, San Jose, CA). The platinum working electrode was fashioned from a Pt crucible and formed onto a Teflon disc. The counter electrode is a plain carbon cloth fabric from AvCarb Material Solutions (Lowell, MA) and the Ag/AgCl reference electrode was purchased from BASi (West LaFayette, IN).

Chemicals. Lithium triflate (96%), 4,4'-dimethyoxydiphenylamine (DMDPA, 99%), di-p-tolylamine (DPTA, 97%), and tetrabutylammonium hexafluorophosphate (98%), were purchased from Sigma Aldrich. All chemicals were used out of the bottle without further purification. DMDPA and DPTA solutions were prepared using 1 mM LiOTf solution in acetonitrile. d-14 di-p-tolylamine was prepared according to Procedure A from Tlili et al.[1] and characterized via ^{1}H NMR and high-resolution mass spectrometry.

Electrochemical Methods. Potentials were controlled across the electrodes using a potentiostat (WaveNow, Pine Research Instrumentation, Durham, NC). A metal contact rests on the rotating electrode surface to maintain electrical contact between the three electrodes. Bulk electrolysis experiments are set to run with zero potential applied to the working electrode for 30 seconds followed by application of an oxidation potential for 30 seconds. This is done three times to determine if the observations are reproducible within a single run.

CID MS/MS spectrum of protonated DMDPA. 100 μM DMDPA was sprayed at an infusion rate of 10 μL/min directed toward the surface of the Pt waterwheel electrode rotating at 1.0 rev/s. An oxidizing potential of 4.0 V was applied to the working electrode. After isolating the *m/z* 230.12 mass peak with a 1.0 isolation width, the ions were bombarded with N_2 gas molecules at normalized collision energy of 25. The peak at 197.3759 is a known background peak.

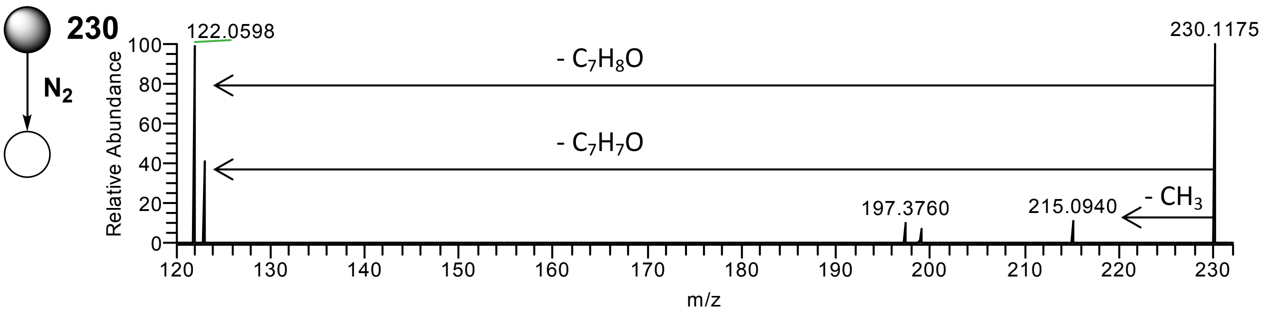

Figure S1. Collision-induced dissociation (CID) MS/MS spectrum of *m/z* 230.1175.

CID MS/MS spectrum of DMDPA radical cation. 100 μM DMDPA was sprayed at an infusion rate of 10 μL/min directed toward the surface of the Pt waterwheel electrode rotating at 1.0 rev/s. An oxidizing potential of 4.0 V was applied to the working electrode. The custom spray probe delivered DMDPA and simultaneously directed the reaction mixture to the mass spectrometer. After isolating the *m/z* 229.11 mass peak with a 1.0 isolation width, the ions were bombarded with N_2 gas molecules at normalized collision energy of 25.

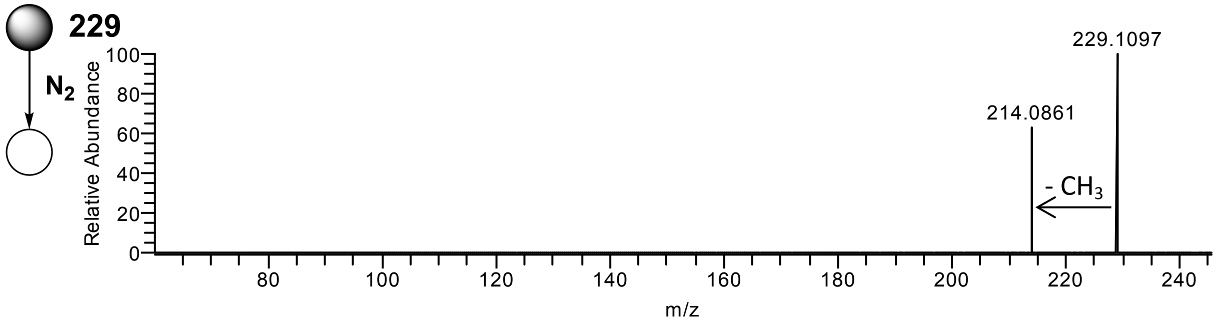

Figure S2. Collision-induced dissociation (CID) MS/MS spectrum of *m/z* 229.1097.

CID MS/MS spectrum of DMDPA nitrenium ion. 100 μM DMDPA was sprayed at an infusion rate of 10 μL/min directed toward the surface of the Pt waterwheel electrode rotating at 1.0 rev/s. An oxidizing potential of 4.0 V was applied to the working electrode. After isolating the *m/z* 228.11 mass peak with a 1.0 isolation width, the ions were bombarded with N_2 gas molecules at normalized collision energy of 25.

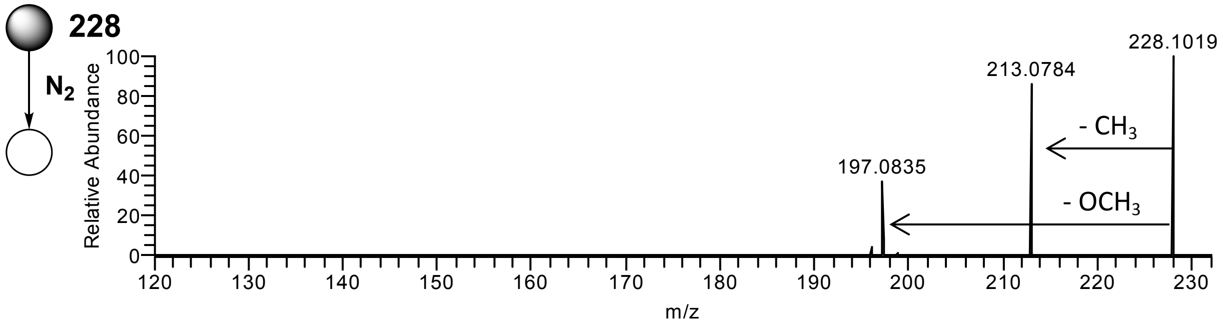

Figure S3. Collision-induced dissociation (CID) MS/MS spectrum of *m/z* 228 .1019.

CID MS/MS spectrum of protonated DPTA. 100 μM DPTA was sprayed at an infusion rate of 10 μL/min directed toward the surface of the Pt waterwheel electrode rotating at 1.0 rev/s. An oxidizing potential of 4.0 V was applied to the working electrode. After isolating the *m/z* 198.13 mass peak with a 1.0 isolation width, the ions were bombarded with N_2 gas molecules at normalized collision energy of 25.

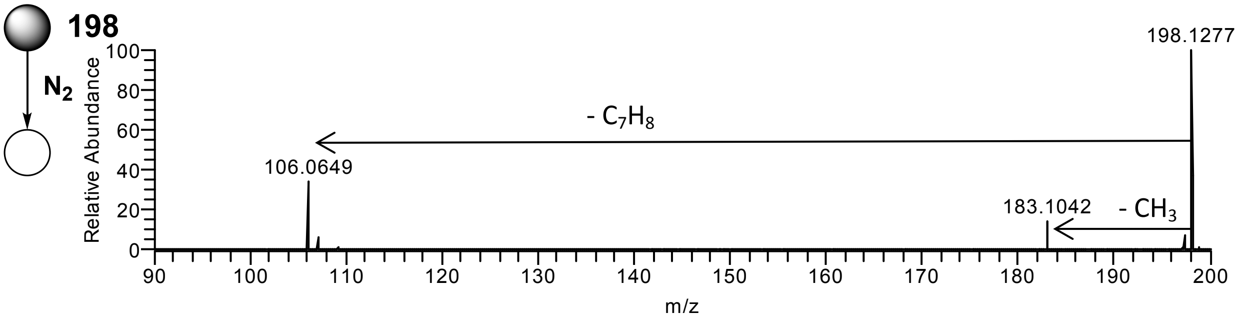

Figure S4. Collision-induced dissociation (CID) MS/MS spectrum of *m/z* 198.1277.

CID MS/MS spectrum of DPTA radical cation. 100 μM DPTA was sprayed at an infusion rate of 10 μL/min directed toward the surface of the Pt waterwheel electrode rotating at 1.0 rev/s. An oxidizing potential of 4.0 V was applied to the working electrode. After isolating the *m/z* 197.12 mass peak with a 1.0 isolation width, the ions were bombarded with N_2 gas molecules at normalized collision energy of 25.

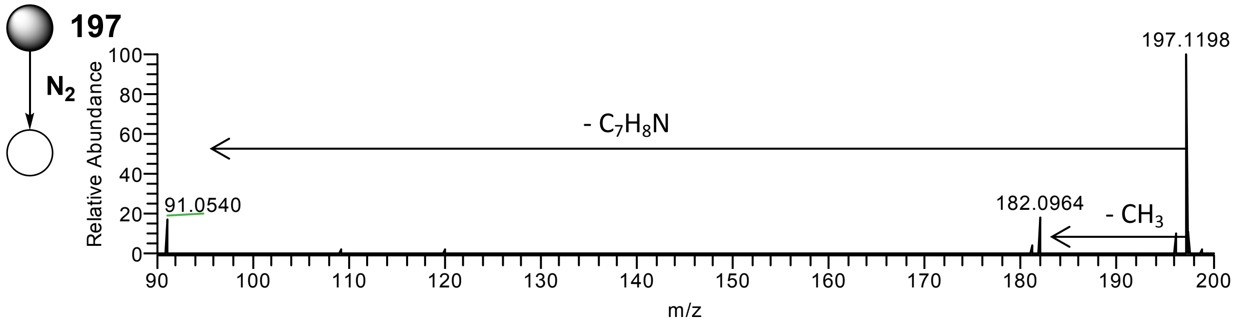

Figure S5. Collision-induced dissociation (CID) MS/MS spectrum of *m/z* 197.1198.

CID MS/MS spectrum of DPTA nitrenium ion. 100 μM DPTA was sprayed at an infusion rate of 10 μL/min directed toward the surface of the Pt waterwheel electrode rotating at 1.0 rev/s. An oxidizing potential of 4.0 V was applied to the working electrode. After isolating the *m/z* 196.11 mass peak with a 1.0 isolation width, the ions were bombarded with N_2 gas molecules at normalized collision energy of 25.

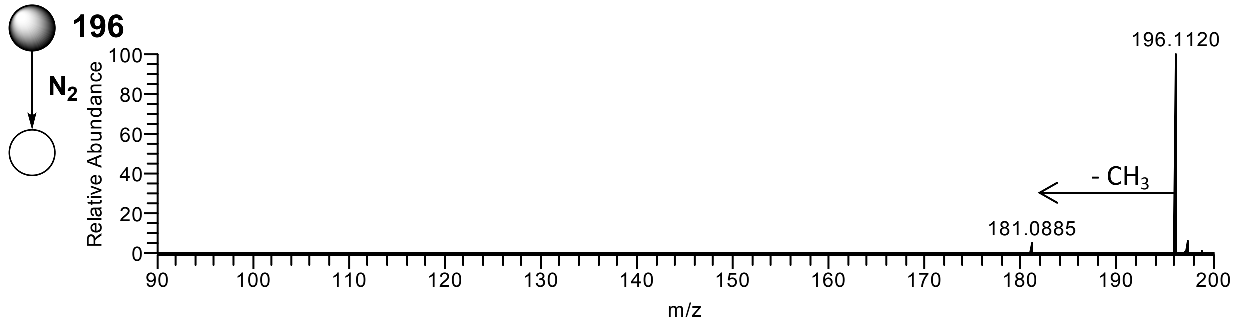

Figure S6. Collision-induced dissociation (CID) MS/MS spectrum of *m/z* 196.1120.

Synthesis of d-14 di-p-tolylamine. The synthesis of (d^{14})-p,p-tolylamine was adapted from an existing literature procedure[1]. After standard cycles of evacuation and backfilling with nitrogen, an oven-dried Schlenk flask with magnetic stir bar was charged with (d^7)-4-iodotoluene (106.1 mg, 0.47 mmol), CuI (26.7 mg, 0.14 mmol), and K_3PO_4 (121.1 mg, 0.47 mmol). It was then transferred into a glovebox where $LiNH_2$ (21.4 mg, 0.93 mmol) was added. Anhydrous, degassed DMF (1.0 mL) was added and the flask was sealed under nitrogen. It was stirred while heating at 130°C for 23 hours. The flask was cooled to room temperature, and 5 mL of DCM were added. The reaction mixture was filtered through celite and washed with further DCM. The filtrate was washed twice with water and the organic layer was then extracted five times with DCM. The organic layers were collected and dried over Na_2SO_4 then the solvent was evaporated *in vacuo*. The crude product was purified by silica gel chromatography using a 80:20 hexane:ethyl acetate eluent. The product, (d^{14})-p,p-tolylamine was isolated as a white powder (20.6 mg, 41% yield). ^{1}H NMR (400 MHz, $CDCl_3$): δ 5.5 (br s, 1H, NH).

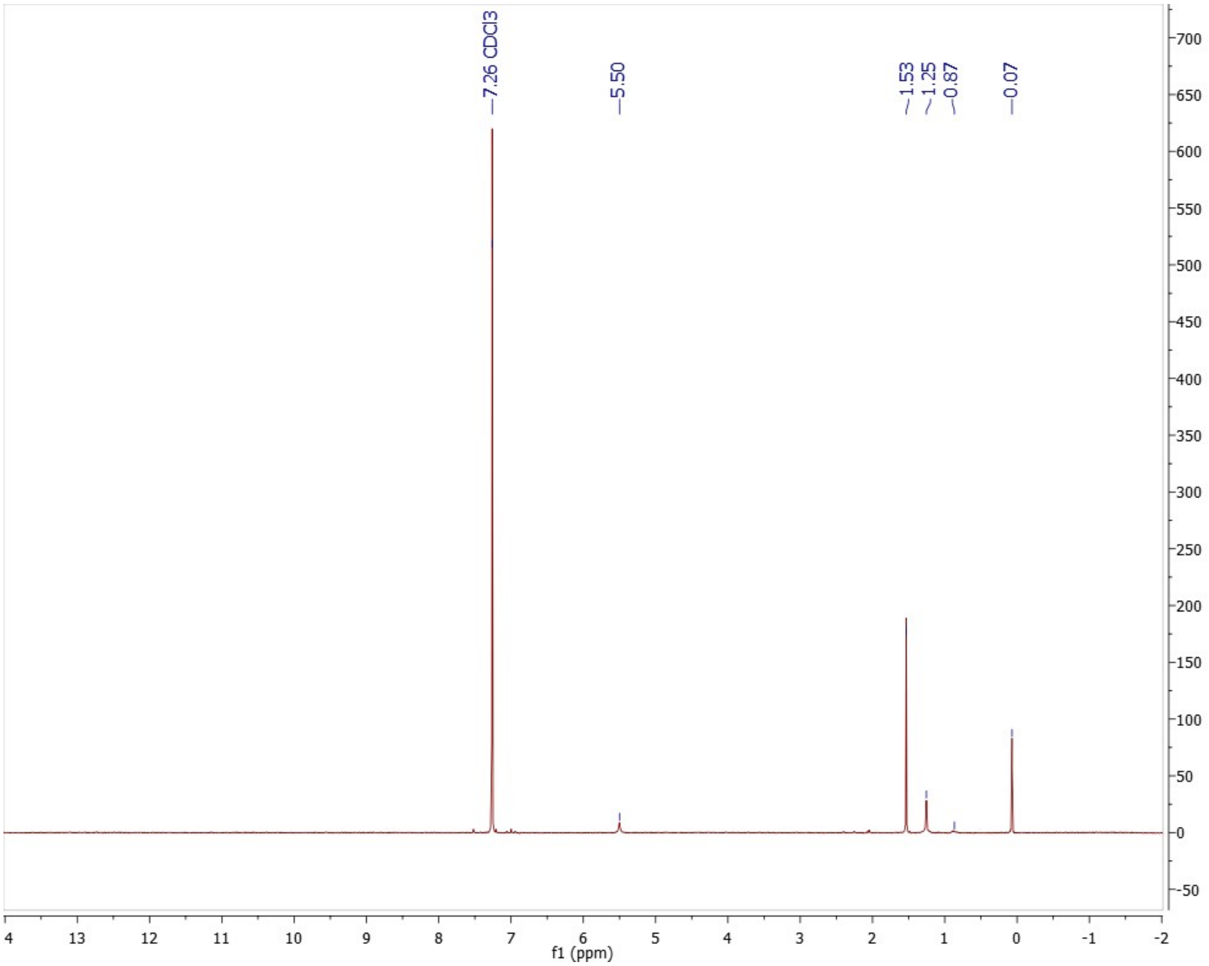

Figure S7. d-14 di-p-tolylamine ^{1}H NMR.

Determination of DPTA ECMS product. A solution of 5 mM d-14 DPTA and 1 mM LiOTf in acetonitrile was held in the reservoir of the previously described setup. A Pt waterwheel electrode rotating at 1.0 rev/s was immersed in the bath. An oxidizing current of 100 mA was applied to the working electrode for 30 seconds. 1 mM LiOTf in acetonitrile was sprayed at an infusion rate of 5 μL/min at the surface of the working electrode to desorb the thin film from the surface for analysis. While the proteo nitrenium and elimination products have the same *m/z* (Scheme S1), the deutero nitrenium and elimination products differ in *m/z* by 1.0063 amu, allowing for differentiating between the two possible products.

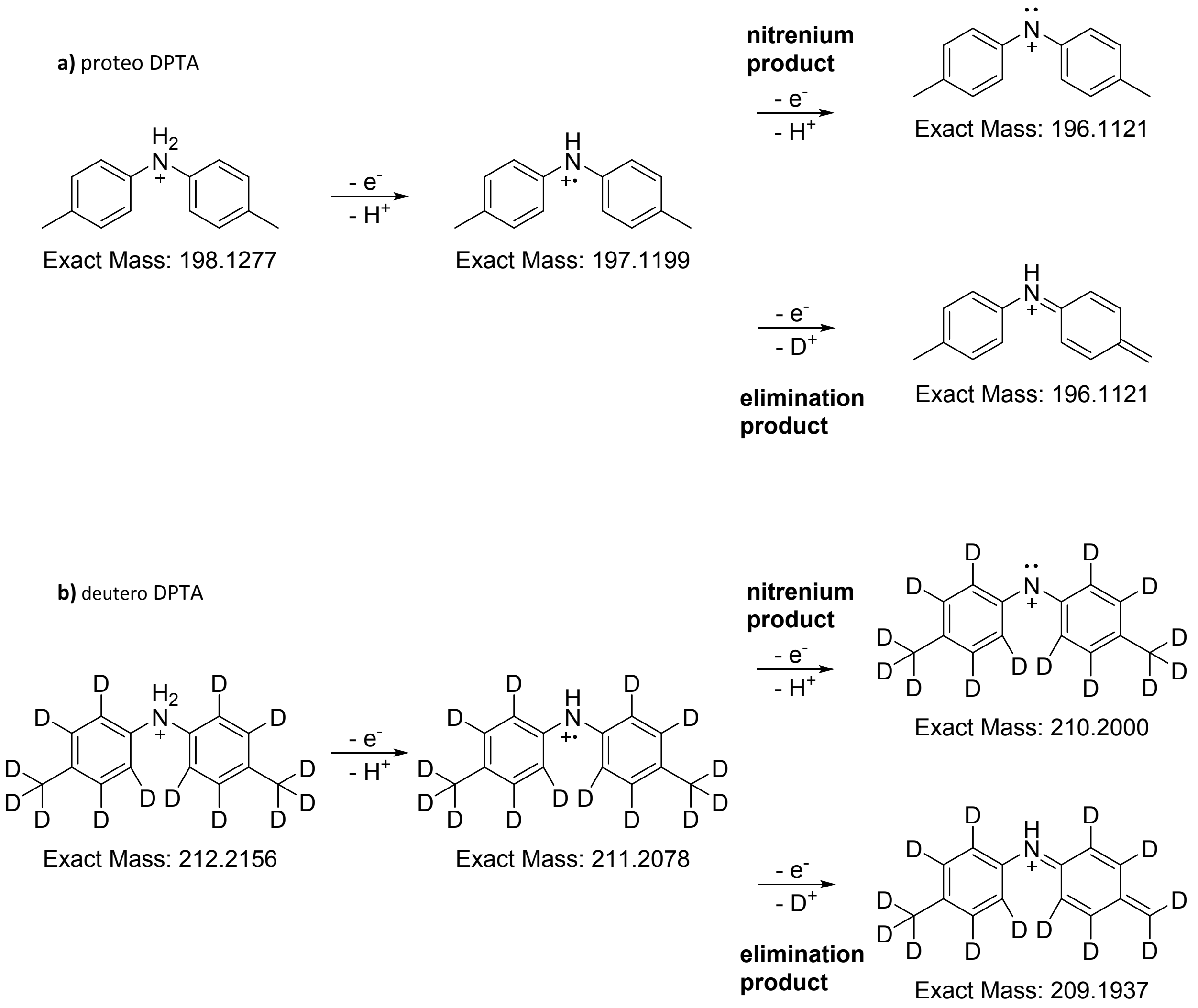

Scheme S1. The competing pathways for electrooxidation of DPTA. a) The mechanism for the formation of the nitrenium ion and the competing elimination product of DPTA; and b) The mechanism for the formation of the nitrenium ion and the competing elimination product of d-14 labelled DPTA.

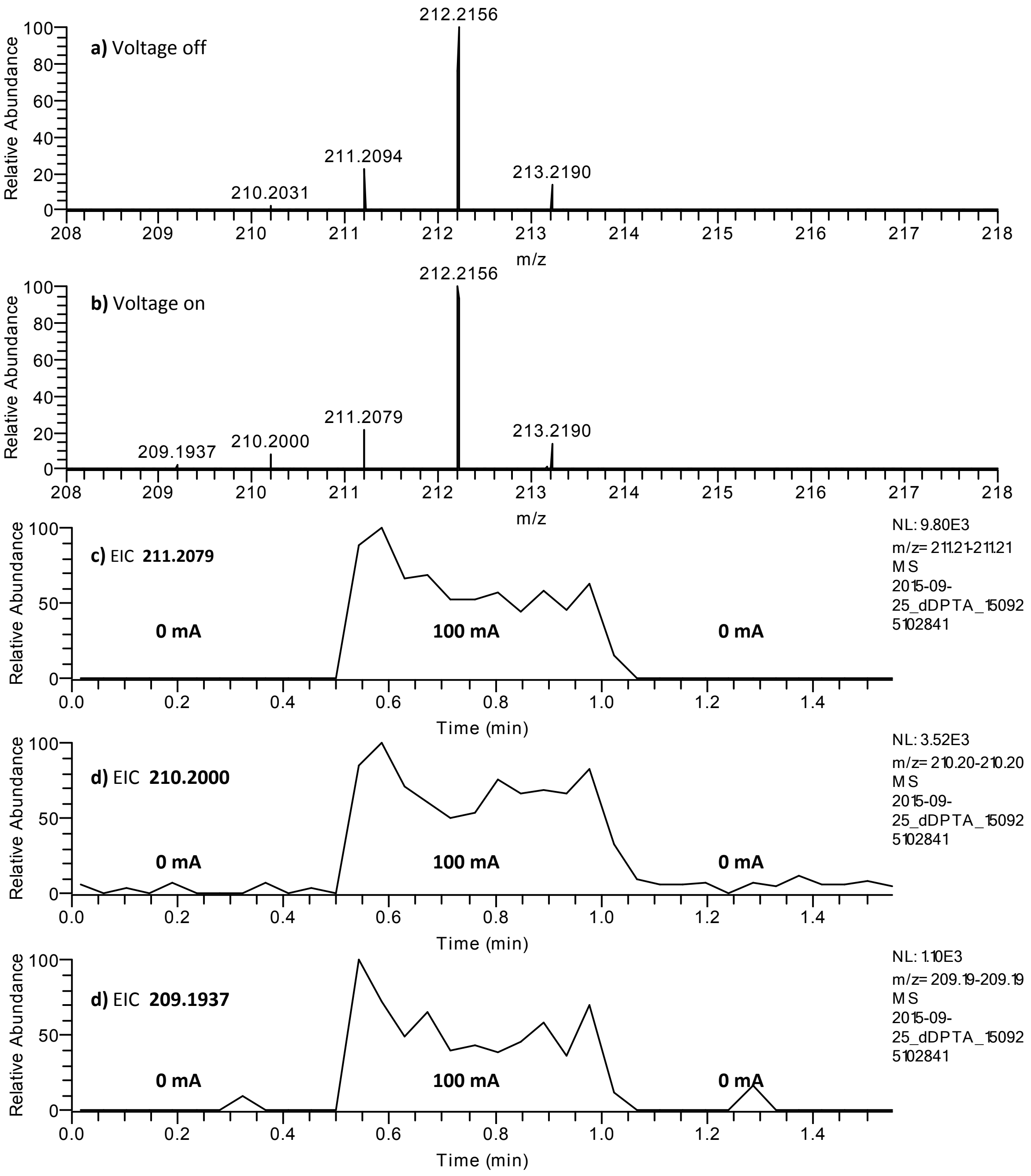

Figure S8. Positive-ion mode mass spectra of d-14 DPTA. a) 0.0 V applied to the working electrode; b) 4.0 V applied to the working electrode; c) EIC for the 211.2079 peak as a function of the applied potential; d) EIC for the 210.2000 peak as a function of the applied potential; and e) EIC for the 209.1937 peak as a function of the applied potential.

References

(1) Tlili, A.; Monnier, F.; Taillefer, M. *Chem. Commun.* **2012**, *48* (51), 6408.
